# Auranofin Alleviates Cognitive Deficit Induced by Cladribine in Experimental Rats

**DOI:** 10.1002/alz.085721

**Published:** 2025-01-09

**Authors:** Khadga Raj Aran

**Affiliations:** ^1^ I.S.F College of Pharmacy, Moga India

## Abstract

**Background:**

A purine nucleoside called cladribine has been shown to increase toxic amyloid protein and cause impaired cognition. Auranofin is a gold(I)‐containing drug with anti‐inflammatory, antioxidant, anti‐apoptotic, anti‐amyloidogenic, and neuroprotective properties. The goal of the current study was to find out the neuroprotective effects of auranofin against cladribine‐induced Aβ accumulation associated with AD‐like symptoms in experimental rats.

**Method:**

1 mg/kg, p.o. Cladribine, followed by 5, 10 mg/kg, i.p. auranofin, was given to Wistar rats for 28 days, and behavioral parameters were performed. On day 29, Wistar rats were sacrificed, and the hippocampus part of the brain was isolated for estimation of pro‐neuroinflammatory markers, biochemicals, neurotransmitters, and molecular pathways analysis.

**Result:**

Rats administrated with cladribine showed elevated cytokine release, oxidative stress, biochemical changes, and a dysbalanced concentration of neurotransmitters. However, auranofin treatment dose‐dependently increased concentrations of antioxidant enzymes decreased the release of proinflammatory cytokines and neurotransmitters and enhanced recognition and spatial memory in rats. Auranofin downregulated tau phosphorylation, Aβ (1–42) levels and regeneration of hippocampal cells. Moreover, the auranofin restoration of the neuroprotective mechanism includes anti‐inflammatory, antioxidant, anti‐apoptotic, anti‐amyloidogenic, and neurotransmitter restoration. Auranofin treatment improved spatial and recognition memory in Wistar rats, restored concentrations of neurotransmitters, antioxidant enzymes and slowed down the release of proinflammatory cytokines in a dose‐dependently way. Additionally, auranofin cleared the accumulation level of Aβ (1–42) and enhanced the Na^+^/K^+^‐ATPase activity in the hippocampal cells. Additionally, the histopathological results showed that auranofin mitigates the neuronal death caused by cladribine in the hippocampus.

**Conclusion:**

We can conclude that auranofin reduced amyloid‐β (1–42), tau phosphorylation levels, and neurotransmitter balance and improved Na^+^/K^+^‐ATPase activity.

